# High performance and gate-controlled GeSe/HfS_2_ negative differential resistance device†

**DOI:** 10.1039/d1ra07276e

**Published:** 2022-01-05

**Authors:** Amir Muhammad Afzal, Muhammad Zahir Iqbal, Muhammad Waqas Iqbal, Thamer Alomayri, Ghulam Dastgeer, Yasir Javed, Naveed Akhter Shad, Rajwali Khan, M. Munir Sajid, R. Neffati, Tasawar Abbas, Qudrat Ullah Khan

**Affiliations:** Department of Physics, Riphah International University 13 Raiwind Road Lahore Pakistan amirafzal461@gmail.com; Nanotechnology Research Laboratory, Faculty of Engineering Sciences, GIK Institute of Engineering Sciences and Technology Topi 23640 Khyber Pakhtunkhwa Pakistan; Department of Physics, Faculty of Applied Science, Umm-Al-Qura University 21955 Makkah Saudi Arabia; Department of Physics & Astronomy, Graphene Research Institute–Texas Photonics Center International Research Center (GRI–TPC IRC), Sejong University Seoul 05006 Korea; Department of Physics, University of Agriculture Faisalabad 38000 Pakistan; Department of Physics, GC University Faisalabad 38000 Pakistan; Department of Physics, University of Lakki Marwat Lakki Marwat KPK Pakistan; Department of Physics, Faculty of Science, King Khalid University P.O. Box 9004 Abha Saudi Arabia; Laboratoire de Physique de la Matière Condensée, Département de Physique, Faculté des Sciences de Tunis, Université Tunis El Manar, Campus Universitaire 1060 Tunis Tunisia; Greater Bay Area Institute of Precision Medicine (Guangzhou), Fudan University Nansha District Guangzhou Guangdong 511458 P. R. China

## Abstract

Transition metal dichalcogenides (TMDs) have received significant attention owing to their thickness-dependent folded current–voltage (*I*_ds_–*V*_ds_) characteristics, which offer various threshold voltage values. Owing to these astonishing characteristics, TMDs based negative differential resistance (NDR) devices are preferred for the realization of multi-valued logic applications. In this study, an innovative and ground-breaking germanium selenide/hafnium disulfide (p-GeSe/n-HfS_2_) TMDs van der Waals heterostructure (vdWH) NDR device is designed. An extraordinary peak-to-valley current ratio (≈5.8) was estimated at room temperature and was used to explain the tunneling and diffusion currents by using the tunneling mechanism. In addition, the p-GeSe/n-HfS_2_ vdWH diode was used as a ternary inverter. The TMD vdWH diode, which can exhibit different band alignments, is a step forward on the road to developing high-performance multifunctional devices in electronics.

## Abstract 2

A novel and astonishing p-GeSe/n-HfS_2_ NDR device shows a high value for the peak-to-valley current ratio in the range of 5.8.

## Graphical abstract

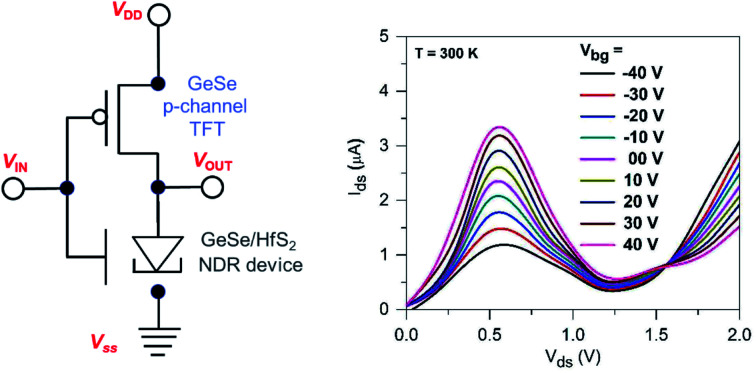

## Introduction

Graphene (Gr), formed from a single layer of carbon atoms, is considered to be a suitable material for use in field-effect transistors (FETs), photodetectors and sensors owing to its zero bandgap.^1–4^ The zero bandgap in Gr has inspired researchers to develop a novel family of materials that possess a suitable bandgap and have analogous features.^5,6^ Beyond the discovery of Gr,^7^ the transition metal dichalcogenides (TMDs) are considered to possess remarkable high performance electronic and optoelectronic characteristics.^8^ TMDs based devices are widely used in different applications, such as photovoltaics, data storage, diodes, and as chemical sensors.^9–13^

Black phosphorus (BP) and molybdenum ditelluride (MoTe_2_) have been widely used in high-performance devices because of their thickness-dependent bandgap and work function, large hole charge carrier densities, and unexpected mobility. Recently, both materials have attracted extraordinary attention owing to their unique optical characteristics.^14–16^ On the other hand, the electronic and optical properties show deterioration because both TMDs are unstable and easily oxidized under ambient conditions.^17^ In contrast, GeSe, an emerging and auspicious p-type transition metal dichalcogenide (TMD), with a unique structure, is considered to be an alternate to BP. The bandgap nature of GeSe changes from indirect (1.08 eV) to direct (1.7 eV) in the bulk compared to the monolayer, respectively.^18–20^ It has significant application potential in FETs, chemical sensors, and photovoltaic devices.^21^

Other widely studied Hf-based layered TMDs, such as HfS_2_ and HfSe_2_, in which the layers are linked by van der Waals forces, have been discovered. The HfS_2_ has an octahedral coordinate structure with an indirect bandgap (1.1–1.2 eV) in the case of the monolayer.^22^ It has broad applications in electronics and optoelectronics because of its narrow bandgap, large and tunable work function, and huge mobility.^23–25^ It is clear from the literature that the device applications of HfS_2_ are quite limited. Contact resistance also plays a vital role in the performance of the devices; a genuine issue is presented when the work function is not the same as that of the TMDs materials and metal contacts.^26,27^ If there is contact resistance between the metal contact and the TMDs materials it is difficult to achieve a high performance in electronics and optoelectronics. By stacking the TMDs materials, a compact system is developed called the van der Waals heterostructures (vdWHs), which offers a novel platform to develop nano-devices.

The nano devices based on 2D-TMDs vdWHs have been efficiently used in FETs,^28^ sensors,^29^ data storage devices,^30^ photodetectors,^31–34^ integrated circuits,^35^ energy storage,^36^ amplifiers,^37^ inverters,^38^ spin-field effect transistors,^39^ water splitting,^40^ and diodes.^38,41^ Heterostructures and homojunction type devices have been designed by the doping of TMDs materials (chemically and electrostatically), and Fermi-level pinning. These techniques are not suitable for use in a high-performance device. The performance of the devices decreased with the passing of time. Secondly, the performance of the TMDs based nano devices is also controlled by controlling the Schottky barrier height (*ϕ*_B_) of the metal–TMDs junction.^38,42^ The *ϕ*_B_ is considered to be a key parameter to control the performance of nano devices.

Hence, the 2D-TMDs vdWHs multifunctional devices with extraordinary performances, such as negative differential resistance (NDR) diode type devices and a high value of peak-to-valley current ratio, have yet to be realized. In this research, we fabricated advanced and unique GeSe/HfS_2_ vdWHs for multiple applications, such as NDR diode type devices and broadband photo-detecting. Each material (p-GeSe and n-HfS_2_) was characterized using different metal contacts to estimate the low resistance electrode and high mobility. The high peak-to-valley current ratio values were estimated at room temperature and explain the tunneling and diffusion currents using the tunneling mechanism. These high gate-modulated NDR characteristics, with an extraordinary peak-to-valley current, represent an outstanding potential in electronics, which are likely to be essential when developing highly efficient multi-valued logic applications.

## Materials and methods

### Device fabrication

Nano-flakes of the TMDs material (p-GeSe and n-HfS_2_) were obtained by mechanical exfoliation. The Scotch tape method was used to exfoliate the materials. The dry transfer method was used to fabricate the NDR devices. A compound microscope was used to observe and estimate the thickness of the p-GeSe flakes. After selection of the GeSe flake, a polydimethylsiloxane (PDMS) stamp was used to make the n-HfS_2_ flake a suitable size and it was then placed onto a p-GeSe flake with a micro-aligner stage. The electrode pattern was designed, and a thermal evaporation system was used for the metal contacts of (Pd/Au, Ni/Au, Cr/Au, and Ag/Au: 6/60 nm) and (Sc/Au, Al/Au, Ti/Au, Pt/Au: 6/60 nm) for p-GeSe and n-HfS_2_, respectively. For the lift-off process, acetone was used.

All of the measurements (electrical) were performed in a vacuum box with a Keithley-2400 and Keithley-6485.

## Results and discussion

**Fig. 1:**
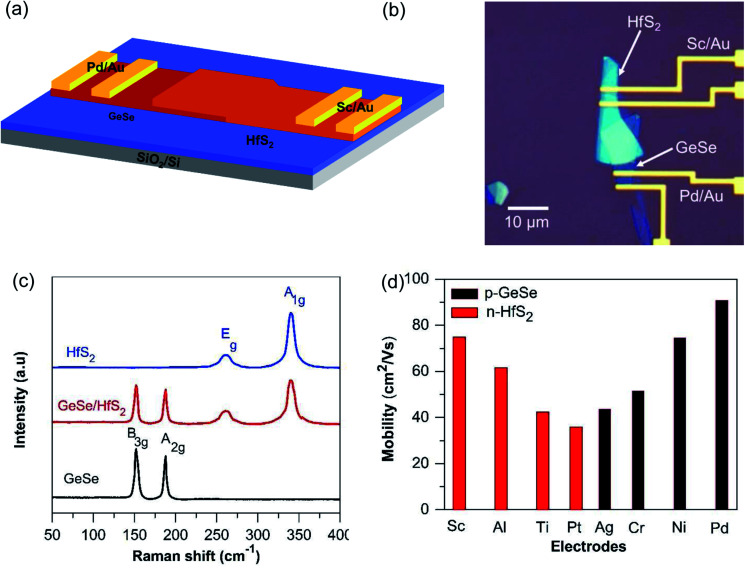
(a) Schematic diagram of the GeSe/HfS_2_ broken band gap p–n heterojunction device. (b) Optical image of the p-GeSe/n-HfS_2_ broken band gap p–n junction. (c) Raman spectroscopy of each flake (GeSe and HfS_2_) and the heterojunction (GeSe/HfS_2_) device, respectively. (d) Mobility of the p-GeSe and HfS_2_ TMDs with different electrodes.

The p-type GeSe was exfoliated and moved onto the substrate (p-Si/SiO_2_ = 300 nm) with the help of a micro-aligner attached to the camera of a microscope. The n-type HfS_2_ was transferred onto the GeSe to form the vdWHs. A schematic diagram is shown Fig. 1a. The optical image of the final device is shown in Fig. 1b. Raman spectroscopy was used for the identification of materials. Fig. 1c shows the Raman spectra of each material (GeSe and HfS_2_) and the heterostructure. Raman spectroscopy was used to determine the nature of the materials. Fig. 1c shows the Raman spectra of GeSe, HfS_2,_ and GeSe/HfS_2_. The peaks that appear at 151.8 and 191 cm^−1^ in the case of GeSe match those observed in the previously published literature.^19,43^ In the case of n-HfS_2_, the Raman peaks appeared at 260 and 337 cm^−1^ and the Raman modes are consistent with previous reports.^44,45^

Atomic force microscopy (AFM) was used to determine the thickness of the p-GeSe and n-HfS_2_ flakes, and these were found to lie in the range of 16 and 28 nm, respectively. The AFM image with height profiles is shown in Fig. S1a and b (ESI†). The electrode Pd/Au and Sc/Au is used for p-GeSe and HfS_2_, respectively, to form the low resistance contacts. The types of charge carriers in p-GeSe and n-HfS_2_ were confirmed by the transfer curve at a constant *V*_ds_ = 0.5 V (Fig. S2a and b†). The *V*_bg_ was swept between ±40 V and measured the output across the source to the drain.

First, we measured the p-GeSe and n-HfS_2_ field-effect transistors with dissimilar metal electrodes and then performed electrical measurements. To estimate the electrical performance, the charge carrier mobility (*μ*_FE_) of p-GeSe and n-HfS_2_ was measured. The *μ*_FE_ of the device was extracted by using the following relationship:^46^1
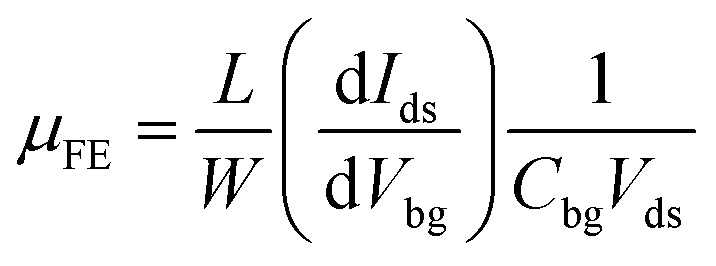


In which, *L* and *W* represent the length and width of the channel respectively, 
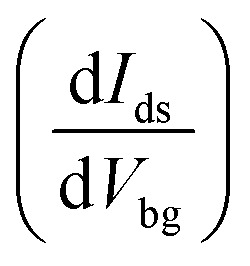
 provides the slopes, and *C*_bg_ is the gate capacitance. In GeSe FET, the estimated *μ*_FE_ were observed to be Pd = 90.7, Ni = 74.5, Cr = 51.4, and Ag = 43.5 cm^2^ V^−1^ s^−1^.^47^ Secondly, the conceived *μ*_FE_ in the case of n-HfS_2_ with Sc, Al, Ti, and Pt were found to be 75, 61.6, 42.5, and 36 cm^2^ V^−1^ s^−1^ (Fig. 1d). In the case of the ohmic contacts (Pd–GeSe/Sc–HfS_2_), a large hole/electron mobility and lower value of the threshold voltage were obtained because of the high/low work function, respectively. The Pd/Sc metals with a high/low work function doped the GeSe/HfS_2_ TMDs materials and improved the charge carrier densities in the channel of the device. Furthermore, the current–voltage (*I*_ds_–*V*_ds_) characteristics were estimated using various contacts to determine the linear and non-linear behavior of the electrodes. Fig. S3† displays the *I*_ds_–*V*_ds_ curves of the GeSe and HfS_2_ with various metal contacts. The metal contact (Pd), which has a high work function, shows a linear behavior (ohmic) with GeSe. Other metals show non-linear curves because of the difference in the work function. The linear behavior of the *I*_ds_–*V*_ds_ curves validate the ohmic behavior because of the low potential barrier between the junction of the metal–TMDs. Furthermore, the *I*_ds_–*V*_ds_ characteristics were also measured with different electrodes (Sc, Al, Ti, and Pt) to find the most suitable electrode for high-performance n-HfS_2_ devices. Primarily, the linear tendency of the *I*_ds_–*V*_ds_ characteristics depends on the difference in the work function between the metal and TMDs materials and potential barrier between them. The energy band diagram of the GeSe with different metals before and after contact between the metals and the GeSe material is shown in Fig. S4.† The values of the work functions, electron affinities and bandgaps of GeSe and n-HfS_2_ were taken from previously reported studies.^41,48–56^

**Fig. 2:**
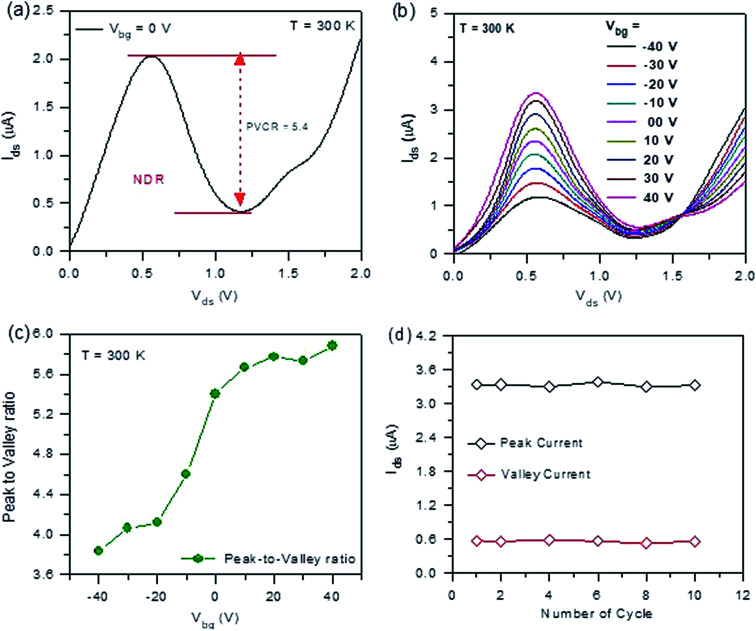
Electrical measurement of the p-GeSe/n-HfS_2_ NDR devices. (a) Current as a function of the bias voltage at *V*_bg_ = 0 V. (b) Change in current at different *V*_bg_ of p-GeSe/n-HfS_2_ NDR devices. (c) Change in the peak to the valley with back gate voltage at room temperature. (d) Change in the peak current and valley current *versus* the number of cycles.

Temperature-dependent electrical measurements were performed to obtain the Schottky barrier height (*ϕ*_B_). Then, we extracted the *ϕ*_B_ between the metals with TMDs by using the standard current model for thermionic emission, given as:2
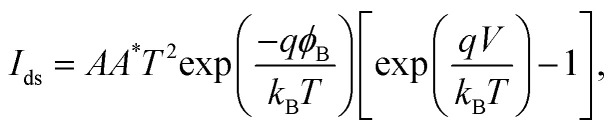
In which, *A* is the area of the junction, A* is the Richardson constant, *q* is the elementary charge (1.6 × 10^−19^ C), *k*_B_ is the Boltzmann's constant (1.38 × 10^−23^ m^2^ kg s^−2^ K^−1^), and *T* is the temperature, respectively. Fig. S5† shows the 
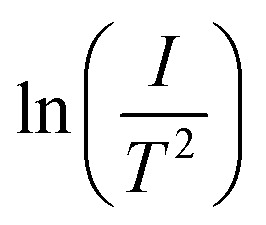
 plotted against 
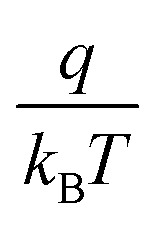
, which is used for the estimation of the *ϕ*_B_ height at metal–TMD junctions. The estimated values of *ϕ*_B_ are 29, 45, 58, and 68 meV for Pd, Ni, Cr, and Ag, respectively. In the case of HfS_2_, the obtained values of *ϕ*_B_ are 41, 52, 60, and 78 meV for Sc, Al, Ti, and Pt, respectively. After the optimization of the most suitable electrode for the Pd–GeSe/Sc–HfS_2_ vdWH heterojunction device, the NDR characteristics were measured. After successfully designing the GeSe/HfS_2_ TMDs vdWH NDR device, the electrical measurement was performed at room temperature. Fig. 2a shows the current–voltage values (*I*_ds_–*V*_ds_) are measured at zero back gate voltages (*V*_bg_). The device demonstrates a high value for the peak-to-valley current ratio (PVCR = 5.4) between 0.5 V and 1.1 V, which is larger than the previously reported literature values.^57,58^ The highest and lowest values of the current are called the peak current (*I*_peak_) and the valley current (*I*_valley_). Fig. 2b shows the gate-dependent *I*_ds_–*V*_ds_ characteristics, which indicates that the peak current (*I*_peak_) is successfully modulated using the back gate voltage (*V*_bg_). When the *V*_bg_ decreases, the *I*_peak_ also decreases. When the *V*_bg_ is tuned from 40 V to −40 V, the Fermi level (*E*_f_) of GeSe moves downward because of the accumulation of charge carriers (holes).^59^

As a result, the energy band bending of GeSe is increased. The *E*_f_ of HfS_2_ is hardly tuned by *V*_bg_ because of the thick GeSe. The shifting of the energy band in the downward direction in GeSe behaves like a potential well at the interface of the heterojunction, which decreases the *I*_peak_. It is difficult to escape the charge carriers from the potential well because of the strong confinement of the electron carriers in the potential well. Therefore, the value of PVCR for the heterojunction was tuned to between 3.83 and 5.87 A/A using the *V*_bg_ (−40 V to 40 V) (Fig. 2c). To check the stability of the *I*_peak_ to *I*_valley_, ten consecutive *I*_ds_–*V*_ds_ sweeps were measured. The estimated values show stable peak- and valley-current values (Fig. 2d).^60^

**Fig. 3:**
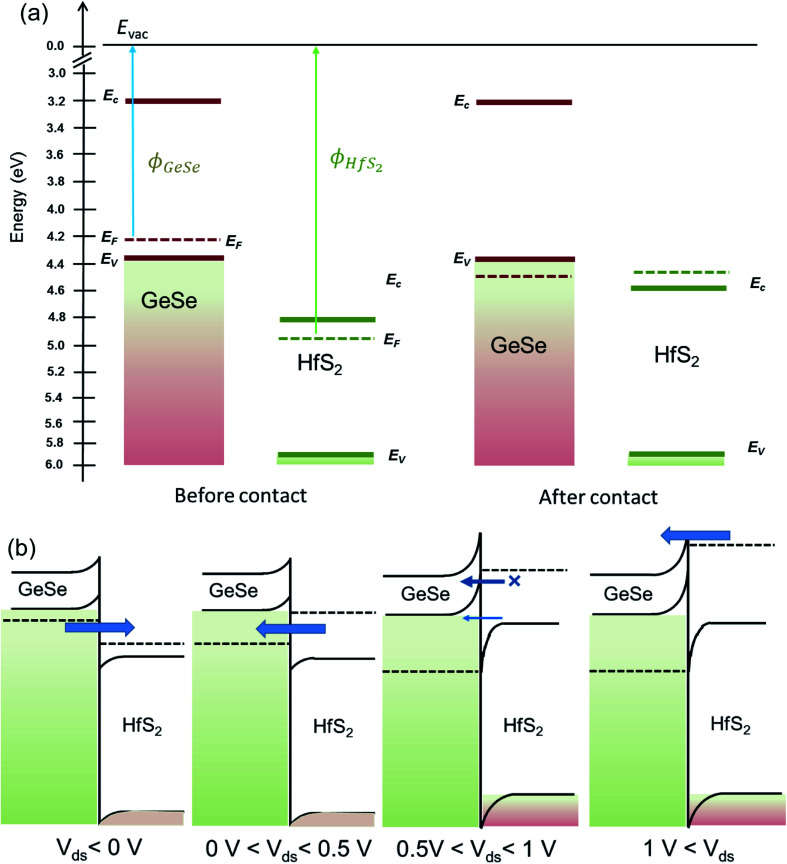
(a) Energy band diagram of the p-GeSe/n-HfS_2_ NDR heterojunction device before contact and after contact at zero back gate voltage. (b) Energy band diagram of the p-GeSe/n-HfS_2_ NDR device at different *V*_ds_.

For a graphical explanation of the NDR mechanism, a band energy diagram is shown in Fig. 3. Fig. 3a shows the band energy diagram of the GeSe/HfS_2_ TMDs vdWH NDR device before and after contact at a zero biased voltage. The *E*_f_of the GeSe and HfS_2_ are aligned after contact at the same level. A type-III bandgap (broken bandgap) was developed between the GeSe and HfS_2_ because the valence band of the GeSe exists above the conduction band of HfS_2_. The current mechanism can be explained based on the diffusion and tunneling current by using the band energy diagram. Fig. 3b shows the energy band diagram of the GeSe/HfS_2_ TMDs vdWH NDR device under a different bias voltage. At a negative bias voltage (*V*_ds_ < 0 V), the tunneling current becomes prominent over the diffusion current. During the negative region, the charge carriers (electron) tunnel from GeSe (filled valence band states) to HfS_2_ (empty conduction band states), which amplifies the current. The tunneling current also becomes prominent between 0 and 0.5 V. In this region, the charge carriers (electrons) tunnel from the conduction band of HfS_2_ to the empty valence states of GeSe, increasing the current. The current is unceasingly enhanced in anticipation of the Fermi level of HfS_2_, which is aligned with the uppermost valence band energy of the GeSe. The overlapping of the filled states in HfS_2_ with unoccupied states in GeSe gives rise to the maximum tunneling current (*I*_peak_). In the range of 0.5 V < *V*_ds_ < 1 V, the magnitude of the current is decreased because of the reduction in the degree of overlap between the filled and empty states. Therefore, the tunneling current is decreased as the *V*_bias_ is increased, which results from the NDR behavior in the heterojunction device. At a higher bias voltage (*V*_ds_ > 0.5 V), the diffusion current predominantly contributes to the NDR behavior of the GeSe/HfS_2_ TMDs vdWH NDR device. In this region, the electrons are capable of diffusing from HfS_2_ to GeSe by attenuating the potential well, which again increases the current in the device.

**Fig. 4:**
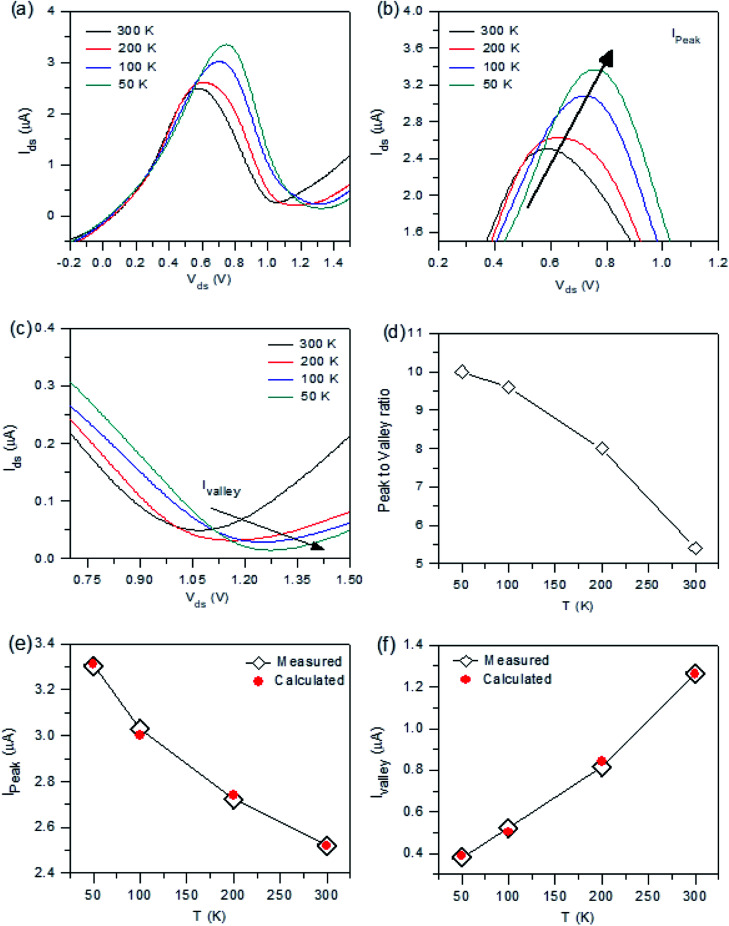
(a) Current–voltage (*I*_ds_–*V*_ds_) characteristics of the GeSe/HfS_2_ NDR device at different temperatures. (b) Change in the valley current with the temperature. (c) Change in the valley current with the temperature of the GeSe/HfS_2_ NDR devices. (d) Change in the peak to valley current with temperature of the GeSe/HfS_2_ NDR devices. (e) Comparison of the measured and calculated value of the peak current as a function of time. (f) Comparison of the measured and calculated value of the valley current as a function of time.

Furthermore, to check the effect of temperature, the *I*_ds_–*V*_ds_ measurements were performed at different temperatures from 50–300 K (Fig. 4a). We observed that the *I*_peak_ is improved, whereas the *I*_valley_ declined with the falling temperature (Fig. 4b and c). The value of the peak-to-valley current was enhanced from 5.8 to 10 A/A with the temperature (Fig. 4d). The values of the peak voltage and valley voltage were shifted towards positive values. To confirm the highest values (Table S1†) of the peak and valley current, the analytic NDR device model was used. The *I*_ds_–*V*_ds_ characteristics were also measured using this model and the peak-to-valley ratio was estimated. The total current (tunneling and diffusion current) is calculated by using the following equation:^52^3
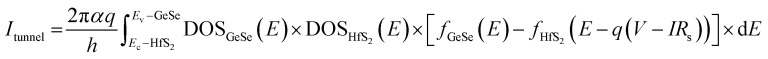
4
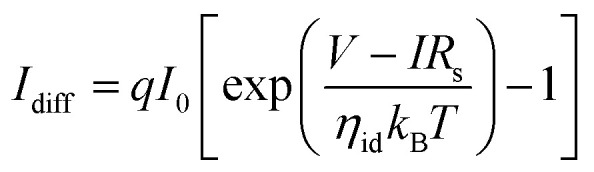


In which *α*, *E*_v_ − GeSe, *E*_c_ − HfS_2_, DOS_GeSe_(*E*), DOS_HfS_2__(*E*), *f*_GeSe_(*E*), and *f*_HfS_2__(*E*) represents the screening factor, the highest valence band energy in GeSe, the lowest conduction band energy in HfS_2_, the density of states and the Fermi–Dirac distribution functions of GeSe and HfS_2_, respectively. Fig. 4e and f show the measured and calculated values, which demonstrate the consistency of the results. The measured value is obtained from the experimental data. The calculated value is conceived from the theoretical *I*_ds_–*V*_ds_ curves estimated using the analytical model.

Basically, most of the *I*_peak_ is primarily occupied by tunnel currents. The *I*_peak_ looks to be allied with the density of states in the CB and VB of the HfS_2_ and GeSe. Both the current values (*I*_peak_ and *I*_valley_) show different behaviors because the tunneling current is increased and the diffusion current is decreased as the temperature decreases. The parasitic series resistance (*R*_s_) was also determined using the analytic model to enable accurate analysis. The *R*_s_ is the contact resistance between the contact and TMD materials. The metal–semiconductor (MS) junction resistance is enhanced at a lower temperature because of the decline in the n-type charge carriers, which leads to an enhanced depletion width at the MS junction. Therefore, a high voltage value is required to operate the NDR device at a lower temperature.^61,62^ We measured multiple devices of the same thickness to check the self-consistency (Fig. S6†). The effect of the thickness is also measured, as shown in Fig. S8.† In the case of a few layers, the device shows a linear behavior because of direct tunneling. Finally, an innovative ternary inverter has been designed, which is the elementary building block of multivalued logic applications. This ternary inverter is fabricated by using the GeSe/HfS_2_ TMDs vdWH. In most thin-film transistors (TFT), the BP was used as p-channel materials that oxidize in the ambient environment and decrease the performance of the devices.^52^

**Fig. 5:**
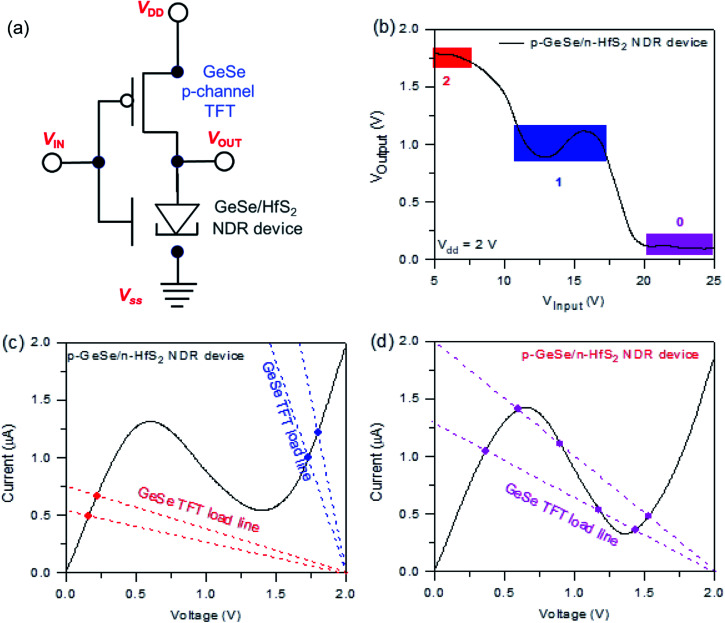
Ternary inverter (p-GeSe/n-HfS_2_ NDR) device: (a) an analog circuit representation of the ternary inverter p-GeSe/n-HfS_2_ NDR device. (b) Output *versus* input characteristics of the ternary inverter p-GeSe/n-HfS_2_ NDR device. (c and d) Load line analysis under different bias conductions for the GeSe/HfS_2_ ternary inverter device.

To overcome this critical issue, we used p-GeSe as a p-channel material. The entire resistance (*R*) in GeSe could be controlled by *V*_bg_. The source and back gate electrodes were used as a supply and input voltage, respectively. The electrode on HfS_2_ is connected to the ground. The output voltage is measured across the middle-shared electrode. Fig. 5d shows the change in *V*_OUT_ as a function of *V*_IN_ at fixed *V*_DD_ = 2 V from 5 to 25 V. We observed three distinct states that are mentioned as state-2, state-1, and state-0. At state 2, the output voltage is greater than 1.75 V, which appears at 4.9 V< *V*_Input_ < 7.9 V. In state-1 and 0 the output voltage is around 0.85 and 0.22 V, respectively (Fig. 5d). Load line circuit analysis was performed to explain the working of the inverter. The point of intersection between the two curves indicates the point of operation of the circuit (Fig. 5c and d).

During the low voltage range, the load resistor offers a path with a small resistance between the source and drain. In this state, the input voltage is higher than the threshold voltage and a high value of the output voltage is obtained, which is almost equal to the *V*_DD_. When a high voltage is applied at the input terminal, the GeSe TFT is turned off and the resistance decreases between the output terminal and the ground. At an intermediate voltage, the device works as an NDR device. The ternary device based on GeSe/HfS_2_ TMDs provides an opportunity to develop multi-valued logic applications.

## Conclusion

In this work, we designed a novel and innovative p-GeSe/n-HfS_2_ TMDs vdWH diode type NDR device. An astonishing peak-to-valley current ratio (≈5.8) was conceived at 300 K. The NDR device was also measured at different back-gate voltages and the NDR behavior was successfully modulated. The performance of the NDR device was also measured at different temperatures. From an application point of view, the p-GeSe/n-HfS_2_ vdWH) diode was used as a ternary inverter.

## Conflicts of interest

The authors declare no conflicts of interest.

## Author contributions

A. M. Afzal performed the experimental work and wrote the manuscript. M. Z. Iqbal, M. W. Iqbal, T. Alomayri, G. Dastgeer, Y. Javed, N. A. Shad, R. Khan, M. M. Sajid, R. Neffati, T. Abbas, and Q. U. Khan helped with the experimental work.

## Supplementary Material

RA-012-D1RA07276E-s001
