# Supplementary material for: High performance and gate-controlled GeSe/HfS2 negative differential resistance device
Source: RSC Adv. 2022 Jan 5;12(3):1278–86. doi: 10.1039/d1ra07276e (PMC8979185; doi:10.1039/d1ra07276e)
Supplement: RA-012-D1RA07276E-s001 [file RA-012-D1RA07276E-s001.pdf]

Electronic Supplementary Material (ESI) for RSC Advances.
This journal is © The Royal Society of Chemistry 2021

Supplementary Data

High Performance and Gate-controlled GeSe/HfS_2 Negative Differential Resistance Device

Amir Muhammad Afzal*[1], Muhammad Zahir Iqbal[2], Muhammad Waqas Iqbal[1], Thamer Alomayri[3], Ghulam Dastgeer[4], Yasir Javed[5], Naveed Akhtar Shad[6], Rajwali Khan[7] M Munir Sajid[6], R. Neffati[8,9], Tasawar Abbas[1], Qudrat Ullah Khan[10]

[1]Department of Physics, Riphah International University, 13 Raiwind Road, Lahore, Pakistan

[2]Nanotechnology Research Laboratory, Faculty of Engineering Sciences, GIK Institute of Engineering Sciences and Technology, Topi 23640, Khyber Pakhtunkhwa, Pakistan

[3]Department of Physics, Faculty of Applied Science, Umm-Al-Qura University, 21955, Makkah, Saudi Arabia

[4]Department of Physics & Astronomy and Graphene Research Institute-Texas Photonics Center International Research Center (GRI–TPC IRC), Sejong University, Seoul 05006, Korea

[5]Department of Physics, University of Agriculture, Faisalabad, 38000, Pakistan

[6]Department of Physics, GC University, Faisalabad, 38000, Pakistan

[7]Department of Physics Lakki Marwat University, Lakki Marwat, KPK Pakistan

[8]Department of Physics, Faculty of Science, King Khalid University; P.O. Box 9004, Abha, Saudi Arabia.

[9]Laboratoire de Physique de la Matière Condensée, Département de Physique, Faculté des Sciences de Tunis, Université Tunis El Manar, Campus universitaire, 1060 Tunis, Tunisia

[10]Key Laboratory of Optoelectronic Devices and Systems, College of Physics and Optoelectronic Engineering, Shenzhen University, Shenzhen, China

* Corresponding author: amirafzal461@gmail.com

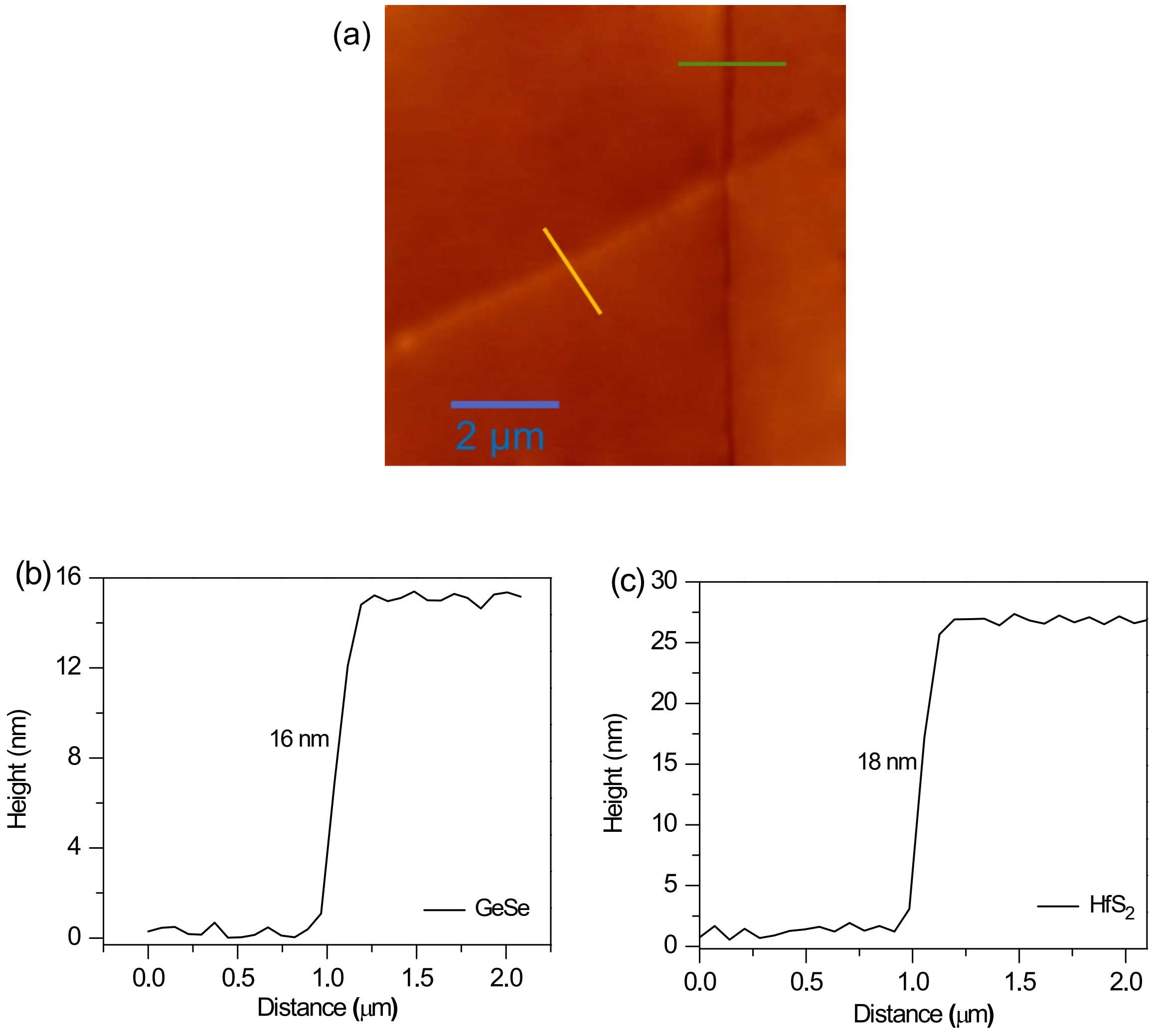

Figure S1. (a) Atomic force microscopy image of GeSe/HfS_2 heterostructure (b) Height profile of GeSe flake. The thickness of GeSe flake is 16 nm (c) Height profile of HfS_2 flake. The thickness of HfS_2 is 18 nm

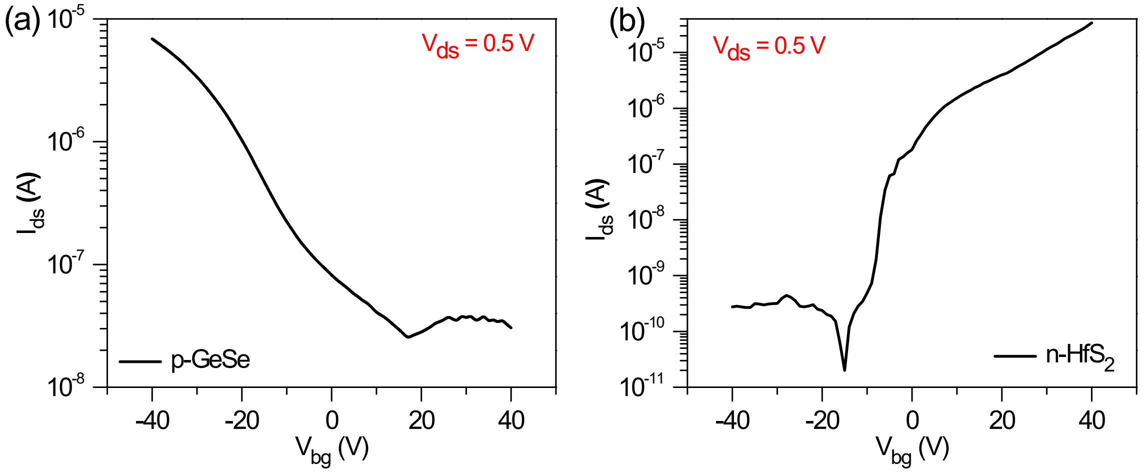

Figure S2. Transfer curves of (a) p-GeSe with Pd/Au (b) n- HfS_2 with Sc/Au

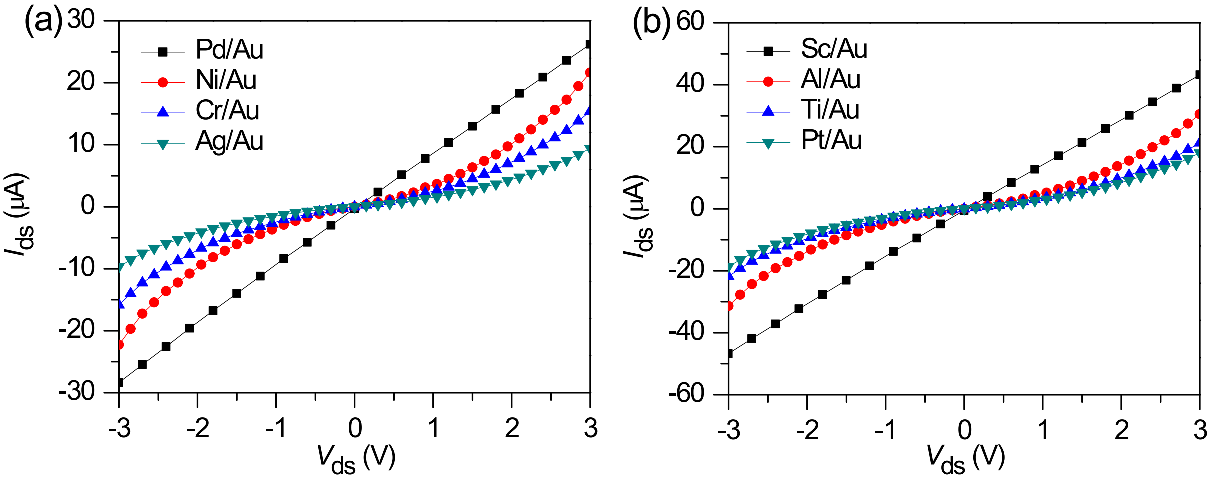

Figure S3. Current-voltage (I_{ds}-V_{ds}) characteristics of (a) p-GeSe with different electrodes (b) n-HfS_2 with different electrodes

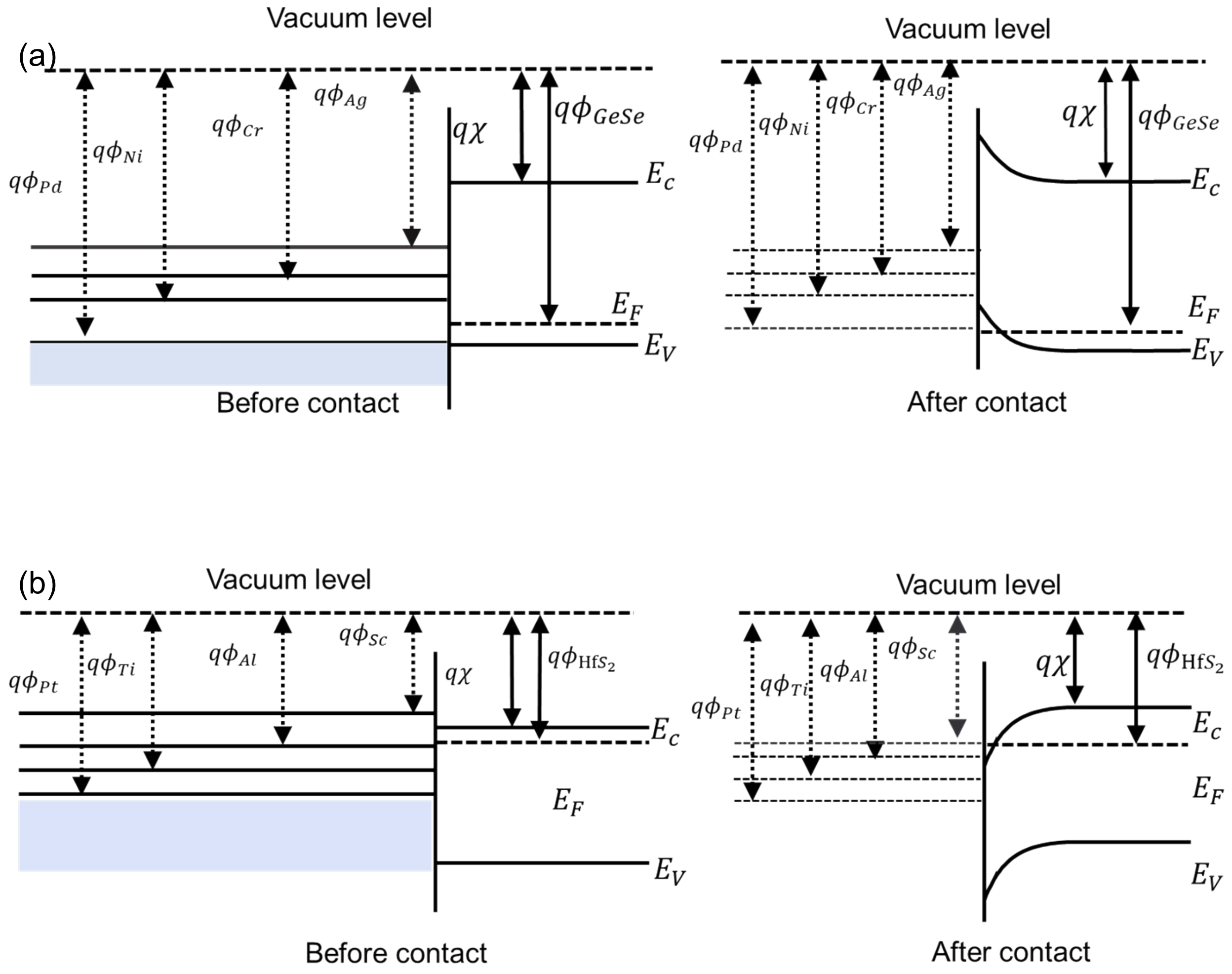

Figure S4. Band energy diagram of metal-TMDs (GeSe and HfS_2) junction before contact and after contact (a) p-GeSe (b) n-HfS_2

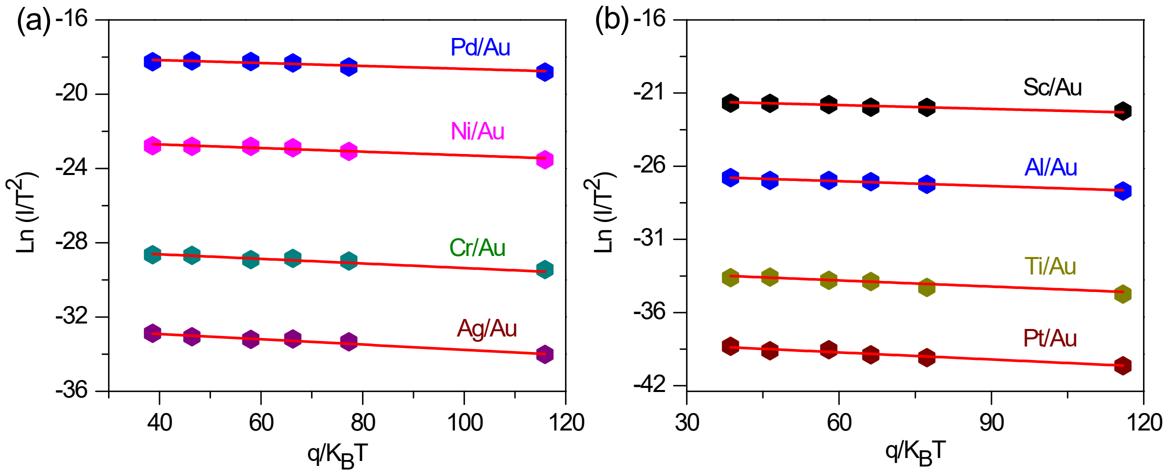

Figure S5. Richardson's plot ($\ln\left(I/T^2\right)$ vs q/K_BT) to calculate the barrier height for (a) GeSe (b) HfS_2

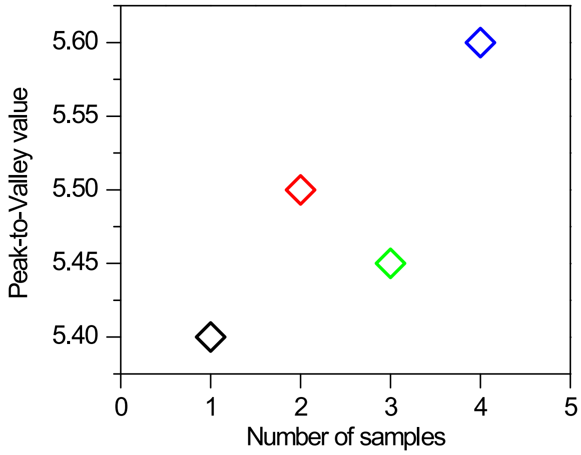

Figure S6. Peak to valley ratio of different samples

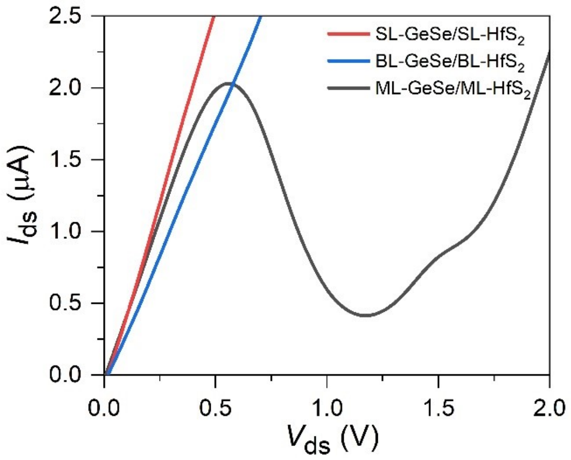

Figure S7. Effect of thickness on the performance of p-GeSe/n-HfS_2 NDR device

Sr. No.	Heterostructure	Peak-to-valley current ratio	Ref.
1	BP/$SnSe_2$	2.8	[1]
2	MoS_2/WSe_2	0.6	[2]
3	MoS_2/WSe_2	1.6	[3]
4	WSe_2/$SnSe_2$	4	[4]
5	BP/$ReSe_2$	3.3	[5]
6	WSe_2	1.5	[6]
7	BP/ReS_2	6.9	[7]
8	GeSe/HfS_2	9.8	This work

Table 1. Comparison of p-GeSe/n-HfS_2 NDR devices with previously reported value.

References

(1) Yan, R.; Fathipour, S.; Han, Y.; Song, B.; Xiao, S.; Li, M.; Ma, N.; Protasenko, V.; Muller, D. A.; Jena, D. J. N. l. Esaki diodes in van der Waals heterojunctions with broken-gap energy band alignment. **2015**, *15* (9), 5791-5798.

(2) Roy, T.; Tosun, M.; Cao, X.; Fang, H.; Lien, D.-H.; Zhao, P.; Chen, Y.-Z.; Chueh, Y.-L.; Guo, J.; Javey, A. J. A. n. Dual-gated MoS2/WSe2 van der Waals tunnel diodes and transistors. **2015**, *9* (2), 2071-2079.

(3) Nourbakhsh, A.; Zubair, A.; Dresselhaus, M. S.; Palacios, T. J. N. l. Transport properties of a MoS2/WSe2 heterojunction transistor and its potential for application. **2016**, *16* (2), 1359-1366.

(4) Fan, S.; Vu, Q. A.; Lee, S.; Phan, T. L.; Han, G.; Kim, Y.-M.; Yu, W. J.; Lee, Y. H. J. A. n. Tunable negative differential resistance in van der Waals heterostructures at room temperature by tailoring the interface. **2019**, *13* (7), 8193-8201.

(5) Srivastava, P. K.; Hassan, Y.; Gebredingle, Y.; Jung, J.; Kang, B.; Yoo, W. J.; Singh, B.; Lee, C. J. S. Multifunctional van der Waals broken-gap heterojunction. **2019**, *15* (11), 1804885.

(6) Paletti, P.; Yue, R.; Hinkle, C.; Fullerton-Shirey, S. K.; Seabaugh, A. J. n. D. M.; Applications. Two-dimensional electric-double-layer Esaki diode. **2019**, *3* (1), 1-7.

(7) Shim, J.; Oh, S.; Kang, D.-H.; Jo, S.-H.; Ali, M. H.; Choi, W.-Y.; Heo, K.; Jeon, J.; Lee, S.; Kim, M.; et al. Phosphorene/rhenium disulfide heterojunction-based negative differential resistance device for multi-valued logic. *Nature Communications* **2016**, *7* (1), 13413. DOI: 10.1038/ncomms13413.
